# The relationship between personality throughout adolescence and social anxiety disorder in young adulthood. A longitudinal twin study

**DOI:** 10.1371/journal.pone.0299766

**Published:** 2024-03-13

**Authors:** Eirunn Skaug, Trine Waaktaar, Svenn Torgersen

**Affiliations:** Department of Psychology, University of Oslo, Oslo, Norway; University of Amsterdam: Universiteit van Amsterdam, NETHERLANDS

## Abstract

This study examined the longitudinal relationship between a range of personality related variables measured throughout adolescence, and social anxiety disorder (SAD) in young adulthood. In addition, we examined to what degree the phenotypic associations between personality and SAD could be attributed to shared genetic and environmental factors, respectively. A total of 3394 twins (56% females), consisting of seven national birth cohorts from Norway, participated in the study. Personality was measured with self-report questionnaires at three times throughout adolescence, and SAD was measured with a diagnostic interview in early adulthood (*M* = 19.1 years, *SD* = 1.2). Correlation and regression analyses were performed to examine phenotypic associations between personality and SAD. We then created four composite scores of personality, in which the personality variables from four different ages throughout adolescence were weighted relative to their importance for SAD. Finally, a series of Cholesky decomposition models were used to examine the underlying genetic and environmental influences on the phenotypic associations between composite scores of personality and SAD. The results showed that especially higher neuroticism, lower extraversion, higher levels of loneliness, and lower levels of resilience, self-efficacy and sense of coherence, were associated with SAD. The phenotypic correlations between composite scores of personality and SAD increased from 0.42 when personality was measured 6–7 years prior to the assessment of SAD, to 0.52 when personality was measured shortly before the assessment of SAD. These phenotypic associations were mainly due to genetic influences, indicating that personality in adolescence predicts SAD in early adulthood due to shared genetic influences rather than having direct ‘causal’ effects on SAD.

## Introduction

Social anxiety disorder (SAD) is characterized by a marked and persistent fear of social situations in which embarrassment or scrutiny from others may occur [1]. Individuals with SAD fear that their way of acting will be negatively evaluated by others, and they often fear that their anxiety symptoms (e.g., sweating, trembling, stumbling over one’s words) will be noticed. Consequently, individuals with SAD often avoid social situations. To be diagnosed with SAD, the fear must be disproportionate to the triggering event, and must cause significant distress or impairment in important areas of functioning, such as in social, academic or occupational domains [1]. SAD is one of the most common occurring mental health disorders in the general population [2], and the onset of this disorder is usually in early adolescence [3]. Hence, it is essential to study SAD in adolescent samples to examine its origins, as well as and risk and protective factors.

Personality may have a large impact on psychosocial functioning, and certain personality traits may thus represent important risk factors in the development of mental disorders [4]. Furthermore, personality and psychopathology are closely related conceptually [5]. Personality refers to our characteristic ways of thinking, feeling and behaving. Similarly, all mental disorders are characterized by disturbances in cognition, emotion regulation and/or behavior [1]. Indeed, numerous studies have examined the relationship between personality and psychopathology among adults [6–8]. Adult personality research has largely focused on the Big Five traits, which include Neuroticism, Extraversion, Openness, Agreeableness, and Conscientiousness [9]. Those who score high on neuroticism tend to experience negative emotions, worry a lot, and are vulnerable to stress. Individuals who score high on extraversion tend to be sociable, talkative, experience positive emotions, and gain energy in social situations. Individuals scoring high on openness tend to be imaginative, intellectually curious, behaviorally flexible, and like to try new things. Furthermore, agreeable individuals tend to be trusting, altruistic, empathic, and care about others. Lastly, those who score high on conscientiousness tend to be thoughtful, well-organized, and goal directed.

Regarding SAD, this diagnosis has been particularly associated with high neuroticism and low extraversion, but also low conscientiousness in adult samples [7, 10]. Of note, this pattern of personality traits is not unique for SAD, but it also found for other common mental disorders such as depression and substance use disorders [7, 11]. Studies on the relationship between personality and anxiety disorders in child and adolescent samples indicate that a similar pattern of personality traits is associated with anxiety in childhood and adolescence (i.e., a pattern of primarily high neuroticism, but also low extraversion and low conscientiousness). Although most studies are cross-sectional, prospective studies of adolescent samples have demonstrated that these personality traits predict subsequent anxiety and depression diagnoses and symptoms [12–16]. Of note, most of these studies have focused on neuroticism and extraversion, while fewer studies have examined conscientiousness.

Beyond the Big Five traits, other personality related characteristics have also been associated with SAD. Loneliness is commonly defined as a negative emotional response to a perceived discrepancy between the desired and actual quantity or quality of social relationships [17]. Of note, empirical studies have found the rank-order of loneliness to be as stable as the rank-order of other personality characteristics, supporting a trait-like nature [18]. It has been hypothesized that loneliness leads to increased sensitivity to signs of rejection and more negative interpretations of others’ behavior, thus making loneliness a potentially important predictor of SAD [19, 20]. However, although numerous cross-sectional studies of child and adolescent samples have reported associations between loneliness and SAD, little is known about how these constructs are related longitudinally [21]. Another concept assumed to play a role in SAD, is self-efficacy. According to social cognitive theory, self-efficacy (i.e., an individual’s belief that he/she will be able to execute the actions necessary to achieve a desired outcome) plays an important role in anxiety arousal [22]. Thus, individuals with lower self-efficacy are believed to develop and maintain higher levels of anxiety as compared to individuals with higher self-efficacy. Although the direction of the effect is unclear, cross-sectional studies of adolescent samples have reported associations between lower self-efficacy and higher levels of anxiety, supporting a proposed relationship between self-efficacy and SAD [23, 24].

Regarding protective factors for SAD (and for psychopathology in general), the concept of resilience has long been a topic of interest. Resilience may be conceptualized as a personality trait that increases the ability to cope with adversity [25]. A meta-analysis of cross-sectional studies examining the relationship between trait resilience and mental health found that trait resilience was negatively associated with mental illness, including anxiety [26]. Although some studies in this meta-analysis included adolescents, most study samples consisted of adults. Related to trait resilience is the concept of sense of coherence. The theory of sense of coherence aims to explain the relationship between stressors, coping and health [27]. Conceptually, sense of coherence may be considered as a personality trait characterized by a relatively stable tendency to view the world more or less predictable, manageable and meaningful [28]. Supporting its trait-like nature, empirical studies have found that sense of coherence shows similar rank-order stability as the Big Five traits [29–31]. Lower sense of coherence is shown to be strongly associated with mental health problems, including anxiety, in both adult and adolescent populations [32–35].

To summarize, there is a growing consensus about the importance of personality in predicting psychopathology [36]. However, most studies on the relationship between personality and anxiety disorders have used adult samples, ‘personality’ is often narrowed down to the Big Five traits in the research literature, and the majority of studies are cross-sectional. Given that the onset of SAD is usually in adolescence, it is essential to study SAD in this age period. Furthermore, longitudinal studies are needed in order to better understand the temporal associations between personality characteristics and SAD. Being able to identify youth at risk of developing SAD before the symptoms have become so severe that they have developed into a clinical diagnosis, may improve the chance of effectively preventing development of the diagnosis and the associated functional impairment. Furthermore, not much is known about the nature of the associations between personality traits and SAD beyond neuroticism. More specifically, genetically informative studies have consistently reported that shared genetic factors account for a substantial amount of the positive association between neuroticism and internalizing disorders, including SAD [37–41]. Studies have also found substantial negative genetic correlations between SAD and extraversion [39, 40], while genetic research on other personality traits and SAD are scarce. Since both SAD [42] and personality traits [43] show substantial heritability, it is important to use research designs that are able to examine to what degree SAD and personality are related due to shared genetic and environmental influences, respectively. For example, associations that are driven to a greater extent by environmental influences are assumed to be more easily modifiable by preventive efforts as compared to associations that are mainly due to shared genetic influences.

By using a longitudinal twin design, this study sought to contribute to a better understanding of the longitudinal relationship between a broad range of personality-related variables throughout adolescence, and SAD in early adulthood. More specifically, we examined the temporal relationship between SAD and several personality-related characteristics, including the Big Five traits, loneliness, self-efficacy, resilience, and sense of coherence. We also examined whether concepts more related to externalizing behavioral tendencies (i.e., impulsivity, conduct problems, and delinquency) were related to SAD. Although SAD is typically characterized by shyness, introversion and behavioral inhibition, a subgroup of SAD seems to be characterized by high impulsivity and disinhibited behavior tendencies [44, 45]. Furthermore, a significant number of adolescents who engage in delinquent behavior also have internalizing mental health problems, such as anxiety symptoms [46]. Hypotheses have been made both of a positive and a negative relationship between delinquency and SAD, and results from empirical studies have yielded mixed results [47, 48]. Impulsivity is often considered a personality trait [49], and both the level of delinquent behavior and conduct problems behavior is shown to be relatively stable in childhood [50], which supports viewing these concepts as trait-like constructs.

Throughout the paper, we will use the word ‘personality’ as a collective term when referring to all independent variables. All these concepts tap into people’s tendencies to think, feel, and behave, and may thus be considered as personality characteristics in a broad sense. In addition, as described throughout in the paragraphs above, empirical studies have found these constructs to have trait-like features. Some concepts are more related to our tendencies to feel (e.g., loneliness), while others tap more into cognition (e.g., self-efficacy) or behavior (e.g., impulsivity).

We had the following specific aims; first, to estimate the phenotypic temporal associations between personality throughout adolescence and SAD in early adulthood; and second, to estimate to what degree the associations are accounted for by genetic and environmental influences.

## Method

### Sample and procedure

Data from the Oslo University Adolescent and Young Adult Twin Project were used for the present study [51, 52]. All twins born in Norway between 1988 and 1994 were invited to participate. This longitudinal project includes three waves of questionnaire data collected throughout adolescence and young adulthood with two years between measurements (12 to 18 years at Wave-1), and data from one face-to-face interview when the twins were around 19 years old (*M* = 19.1, *SD* = 1.2). The study was approved by the Norwegian Data Inspectorate and the Regional Committees for Medical and Health Research Ethics, ref. 2015/4 (19661). American Psychological Association ethical standards were followed in the conduct of the study. Written informed consent was obtained from both the twins and their parents. The process of obtaining informed consents began on March 21st 2006 and ended in mid-August 2006.

In the present study, the self-report questionnaire data from the three waves (in which each wave included data from seven birth cohorts) were rearranged into data from the age of 12–13 years, 14–15 years, 16–17 years, and 18 years and older (i.e., until the time of the interview assessment of SAD). The whole sample consisted of 3394 twins (56% females) from 1716 twin pairs. All twins from both complete and incomplete twin pairs were included in the study. Sample characteristics are presented in Table 1. The majority of those who responded to the questionnaires also participated in the interview (i.e., 76%, 80%, 82% and 87% at age 12–13, 14–15, 16–17 and 18, respectively).

**Table 1:** Twin sample characteristics.

		N single twins	N twin pairs	MZ twin pairs ^a^	DZ twin pairs ^a^
Questionnaire-data					
	12–13 years	856	432	165	259
	14–15 years	1504	767	277	460
	16–17 years	1792	922	329	541
	18 years	1371	782	221	368
Interview-data		2812	1424	543	845

*Note*. MZ = monozygotic; DZ = dizygotic

^a^ Number of complete pairs.

### Zygosity determination

Twin zygosity was determined by a combination of a zygosity questionnaire and gene testing. To validate the zygosity questionnaire, cheek swabbed DNA was drawn from 513 of the 1,006 same-sex twin pairs. Seventeen genetic markers were tested, with an estimated probability of misclassification less than p < 0.0001. The scores on the zygosity scale were analyzed using discriminant analysis and a cutting point for the discriminant score was established based on the results of the gene testing. Those with a discriminant score close to the cutting point were oversampled for DNA tests. It appeared that 14 out of the 513 twin pairs were misclassified according to the discriminant analysis. Correcting for the oversampling, the questionnaire misclassified 2.13% of the same-sex twins. However, as almost all of the misclassified pairs were gene tested, only 0.64% of the same-sex twin pairs are expected to be misclassified (0.45% when including the whole twin sample).

### Data availability

Data for the study was based on the Oslo University Adolescent and Young Adult Twin Project, a longitudinal project that commenced in 2005. The collection of health-related data was pre-approved in 2005 by the Norwegian Data Protection Authority (DPA) under a 20-year clause of individual data protection and subsequent data deletion or anonymization. In light of these legal restrictions, data supporting this article could not be shared at the time of publication. Fully anonymized data underlying this manuscript will be released in January, 2026. From that time, data is planned to be stored in Sikt (Norwegian Agency for Shared Services in Education and Research) and can be accessed in Sikt’s data archive. Data was analyzed using R, version 4.0.3 [53]. The code behind the analyses has been made publicly available at the Open Science Framework and can be accessed at https://osf.io/r3jsh/?view_only=84498ad488ac482ba2b697497c08334c.

### Measures

#### Questionnaire data

In order to maximize the number of scales in the questionnaires, and to reduce dropouts and missing data, the complete scales were abbreviated based on results from a pilot study [51]. That is, the items in the complete scales with the highest item-to-trait correlations across all age groups and across sex were chosen. All scales are presented in S1 Table, and the Cronbach’s alpha for all scales is presented in S2 Table.

*Big Five personality*. The Big Five traits were measured by an abbreviated 40-item version of the Hierarchical Personality Inventory for Children [HiPIC; 54]. The HiPIC is a widely used inventory for the assessment of the Big Five traits in children and adolescents, including emotional stability (reversed neuroticism), extraversion, imagination (corresponding to openness), benevolence (corresponding to agreeableness), and conscientiousness. Items were rated on a 5-point Likert scale ranging from 0 (*not typical*) to 4 (*very typical*). Each personality trait (i.e., neuroticism, extraversion, openness, agreeableness, and conscientiousness) was constructed using the average score of the eight items tapping on each of the five traits.

*Self-efficacy*. Self-efficacy was measured by a 12-item abbreviated version of the Children’s Perceived Self-Efficacy scales [CPSE; 55]. The participants rated how easy they have found it to execute 12 different statements the past 12 months. The scale included items measuring good school habits (e.g., organization of schoolwork, motivation of doing homework), social aptitudes (e.g., capacity to form and maintain friendships, manage interpersonal conflicts) and capacity to resist peer pressure (e.g., drinking alcohol, activities that could get one into trouble). Responses were given on a 5-point Likert scale ranging from 0 (*not easy at all*) to 4 (*very easy*). For each participant an average score was calculated, with higher scores indicating higher self-efficacy.

*Resilience*. Resilience was measured by two resilience scales, including the Resilience Scale [RS; 56] and the Ego-Resiliency Scale [ER89; 25]. Each scale was abbreviated to a 5-item scale. Items from the RS includes statements assessing the degree to which the person follows through with plans, are interested in things, are determined, and believe in him/herself. Items from the ER89 includes statements assessing the degree to which the person enjoy dealing with new situations, like to do new and different things, and whether the person perceive him/herself as having a ‘strong’ personality. Responses were given on a 5-point Likert scale ranging from 0 (*not typical*) to 4 (*very typical*). Average scores were calculated for each scale, with higher scores indicating higher resilience.

*Loneliness*. Loneliness the past year was measured by a 5-item scale, including the 4-item survey version of the R-UCLA Loneliness Scale [57] and one direct measure of loneliness, i.e., “I feel lonely”. Responses were given on a 5-point Likert scale ranging from 0 (*not typical*) to 4 (*very typical*), and average scores were computed with higher scores indicating higher levels of loneliness.

*Sense of coherence*. Sense of coherence the past year was measured by an abbreviated 5-item version of the Sense of Coherence 13-item scale [SOC-13; 58]. The concept of sense of coherence describes the degree to which a person perceives oneself and the world as comprehensible/predictable, manageable, and meaningful. Responses were given on a 7-point Likert scale ranging from 1 (*very often*) to 7 (*rarely/never*). Average scores were computed with higher scores indicating stronger sense of coherence.

*Delinquency*. The participants were asked how often they had committed nine delinquent behaviors over the past 12 months [59, 60]. The scale included items inquiring about physical fights with other persons, stealing, carrying weapon, vandalism, and other rule breaking behaviors such as hanging around at night when the person was supposed to be home. Responses were given on a 4-point Likert scale ranging from 0 (*never*) to 3 (*very often*), and average scores were computed to obtain a total delinquency score.

*Conduct problems*. Conduct problems the past year were measured by the Conduct Problems Scale, a subscale in the Strengths and Difficulties Questionnaire [SDQ; 61, 62]. This 5-item scale includes items inquiring about temper, fights, lying, cheating, and stealing. Responses were given on a 3-point Likert scale ranging from 0 (*not true*) to 2 (*certainly true*). Average scores were computed, with higher scores indicating more conduct problems.

*Impulsivity*. Impulsivity the past year was measured by seven items from the Control Scale (impulsivity reversed) in the brief form of the Multidimensional Personality Questionnaire [MPQ-BF; 63]. The scale included items inquiring about whether the person tend to act on the spur of the moment, tend to plan and organize, and is likely to make up his/her mind through careful reasoning. Responses were given on a 3-point Likert scale ranging from 0 (*false*) to 2 (*true*). Average scores were computed, with higher scores reflecting higher impulsivity.

#### Interview data: Social anxiety disorder

DSM-IV SAD was assessed using the Norwegian Translation of the Mini International Neuropsychiatric Interview [MINI; 64]. A dimensional approach was used to measure SAD (0 = *no SAD*, 1 = *subthreshold SAD*, 2 = *SAD*). Individuals who lacked one or two criteria to meet the full criteria for SAD were considered to have subthreshold SAD. Supporting our dimensional measure, extensive research has shown that differences between people, including differences in psychopathology, are best represented as differences in degree along a continuum rather than qualitative differences in kind [65, 66]. Interrater reliability was assessed based on two raters’ scoring of 55 audiotaped interviews, of which 54 of the recordings were of satisfactory quality to be scored. The intraclass correlation coefficient (ICC) for our dimensional measure of SAD was .97, 95% CI [.96, .98].

### Statistical analyses

#### Phenotypic analyses

To examine the phenotypic associations between SAD in young adulthood and personality at different ages throughout adolescence, we first estimated bivariate correlations. Next, we created four composite scores of personality, in which the personality variables were weighted relative to their importance for SAD. To do so, we performed four regression analyses, with SAD as the dependent variable in each model, and the personality variables at a given age as independent variables (i.e., 12–13 years, 14–15 years, 16–17 years, or 18 years).

#### Biometric analyses

Using data from twins allow us to examine to what degree personality is associated with SAD due to shared genetic and/or environmental influences. The classical twin design allows the variance of an observed phenotype (and the covariance between phenotypes) to be partitioned into genetic and environmental sources of variance (and covariance). This approach relies on comparing the phenotypic similarity between monozygotic (MZ) and dizygotic (DZ) twin pairs. Twins reared together share the same family environment, MZ twins are genetically identical whereas DZ twins share, on average, half of their segregating genes. Genetic influences are inferred by the extent to which the MZ correlation is greater than the DZ correlation. If additive genetic influences (A; the effect of multiple genes that together operate in an additive manner) was the only source to familial resemblance, the DZ correlation is expected to be half the size of the MZ correlation. If the DZ correlation are greater than half the size of the MZ correlation, shared environmental influences (C; any environmental factors contributing to similarity among family members) are inferred. However, if the DZ correlation is less than half of the MZ correlation, non-additive genetic influences (D; interactive genetic effects) are inferred. Of note, D and C effects cannot be estimated simultaneously because they are confounded in the classical twin design. Therefore, we keep C and drop D if the DZ correlation exceed half the size of the MZ correlation. Similarly, we keep D and drop C if the DZ correlation is less than half the size of the MZ correlation. The remaining variance not accounted for by A + C (or A + D) is attributed non-shared environmental influences (E; any factors contributing to phenotypic dissimilarity between family members, including measurement error).

First, univariate twin models were fitted to SAD and the composite scores of personality to examine the influence of genetic and environmental factors on these phenotypes. MZ and DZ correlations for SAD were calculated using polychoric correlations because Pearson correlations will underestimate the association between ordinal variables [67]. Next, we fitted a series of bivariate Cholesky twin decompositions to quantify how much of the phenotypic correlation between the composite scores and SAD that were due to genetic and environmental factors, respectively. The composite score of personality from a given age was included as the first variable, with SAD as the second variable in each model. The bivariate Cholesky twin decomposition partitions the variance in the first variable into genetic and environmental sources and quantify the extent to which these genetic and environmental sources also are contributing to the variance in the second variable. The residual variance in the second variable that is not shared with the first variable is also partitioned into genetic and environmental sources [68].

The biometric analyses were conducted using the structural equation modeling R package OpenMx [69]. All models were fitted to raw data using full information maximum likelihood. SAD was analyzed using a threshold model where a continuous normally disturbed liability is assumed to underlie the observed ordinal variable, and the composite scores were analyzed using models for continuous data. Separate thresholds and means were estimated for males and females to account for sex-differences in mean-level. Competing models were compared using Akaike’s information criterion [AIC; 70], with lower values indicating better model fit.

## Results

### Phenotypic associations

For SAD, measured with a diagnostic interview around the age of 19 (*M* = 19.1, *SD* = 1.2), the mean was 0.18 (*SD* = 0.52). More specifically, 148 (5.3%) and 174 (6.2%) participants were classified as having subthreshold SAD and SAD, respectively. Means and standard deviations for the scales from the questionnaires are presented in S3 Table. We also provide inter-scale correlations and correlation with sex in S4 Table. Overall, the correlations between sex and the study variables were weak (i.e., below .20).

Table 2 presents polyserial bivariate correlations and results from regression analyses, predicting SAD from various personality-related variables measured at four different ages throughout adolescence. Looking at the bivariate associations across all age groups between the Big Five traits and SAD, the results showed that especially higher neuroticism and lower extraversion were associated with SAD. Beyond the Big Five traits, also higher levels of loneliness, and lower levels of resilience, lower self-efficacy, and lower sense of coherence were associated with SAD. Furthermore, it is noteworthy that the bivariate associations were so similar in magnitude, whether personality was measured at age 12–13 years (i.e., 6–7 years prior to the assessment of SAD) or relatively concurrent in time as the assessment of SAD. The beta values in Table 2 are probably underestimated using linear regression when the dependent variable is ordinal. However, as described in the method section, these analyses were conducted to be able to create composite scores of personality in which the variables are given weight depending on their importance for SAD.

**Table 2:** Bivariate correlations between personality and SAD, and results from linear regression analyses, predicting SAD.

	12–13 years		14–15 years		16–17 years		18 years	
Variable	corr	beta	corr	beta	corr	beta	corr	beta
N	.25***	.02	.35***	.15***	.33***	.03	.39***	.11*
E	-.32***	-.06	-.34***	-.08	-.40***	-.16***	-.49***	-.22***
O	-.24***	.01	-.19***	-.01	-.15***	.04	-.22***	.04
A	-.04	.05	-.05	.10**	-.07	.03	-.06	.02
C	-.22***	.00	-.18***	-.08	-.15***	-.05	-.17***	-.08
SEF	-.27***	-.06	-.21***	.07	-.31***	-.06	-.27***	.00
RS	-.29***	-.05	-.24***	.04	-.25***	.05	-.27***	.05
ER	-.28***	-.02	-.28***	-.06	-.32***	-.07	-.35***	-.02
LON	.32***	.15**	.30***	.07	.32***	.01	.33***	.01
SOC	-.18***	-.06	-.24***	-.04	-.29***	-.10**	-.30***	-.07
DEL	.07**	.03	.09	.03	.07	.03	.01	-.01
CON	.02	-.06	.11*	.07	.05	-.01	.06	-.02
IMP	-.03	.00	-.09	-.06	-.15**	-.07*	-.18***	-.06

*Note*. SAD = social anxiety disorder; corr = polyserial bivariate correlation with SAD; beta = standardized beta, predicting SAD from all independent variables; N = neuroticism; E = extraversion; O = openness; A = agreeableness; C = conscientiousness; SEF = self-efficacy; RS = Resilience Scale; ER = Ego Resilience; LON = loneliness; SOC = sense of coherence; DEL = delinquency; CON = conduct problems; IMP = impulsivity.

*p < 0.05

**p < 0.01

***p < 0.001.

### Biometric analyses

We fitted univariate twin models to SAD and the composite scores of personality in order to partition the variances into genetic and environmental influences. SAD showed high genetic effects, with additive genetic influences explaining 59% of the variance. The remaining variance was accounted for by non-shared environmental influences. For the composite scores, heritability estimates ranged from 46% to 61%. Twin correlations, univariate parameter estimates, and fit statistics are presented in S5 Table.

To quantify the extent to which personality in adolescence are related to SAD in young adulthood due to genetic and environmental influences, we fitted a series of bivariate Cholesky decomposition models. More specifically, we partitioned the phenotypic correlations between the composite scores of personality and SAD into genetic and environmental influences. Table 3 presents polyserial cross-trait correlations between the composite scores and SAD. The phenotypic correlations reflect how well SAD can be predicted from personality. As expected, the phenotypic correlations increased somewhat in magnitude as the time interval between the measurements decreased. The patterns of twin correlations indicate that the associations between personality and SAD were mainly due to genetic influences. More specifically, the DZ correlations were not greater than half the size of the MZ correlations, indicating no influence of shared environmental factors. Furthermore, the extent to which the MZ correlations are lower than the phenotypic correlations is indicative of shared influences in the non-shared environment between the predictor variables and SAD. Thus, shared influence of non-shared environmental factors in the association between personality and SAD seem to be negligible when personality was measured at 12–13 years and 14–15 years. From 16–17 years, on the other hand, non-shared environmental influences seem to make a significant contribution to the association between personality and SAD.

**Table 3:** Cross-trait correlations.

	Polyserial correlation with SAD		
Variable ^a^	Phenotypic	rMZ	rDZ
Personality 12–13 years	.42 [.32, .53]	.47 [.33, .61]	.22 [.05, .39]
Personality 14–15 years	.44 [.36, .51]	.43 [.32, .55]	.15 [.02, .28]
Personality 16–17 years	.48 [.41, .55]	.36 [.24, .47]	.10 [-.01, .21]
Personality 18 years	.52 [.45, .59]	.37 [.30, .43]	.14 [.01, .26]

*Note*. 95% confidence intervals in brackets. SAD = social anxiety disorder; Phenotypic = cross-trait correlation without considering twin-pair membership; rMZ = cross-trait correlation within monozygotic twin pairs; rDZ = cross-trait correlation within dizygotic twin pairs

^a^ Composite score of personality, where the personality variables were weighted relative to their importance for SAD.

**Fig 1:**
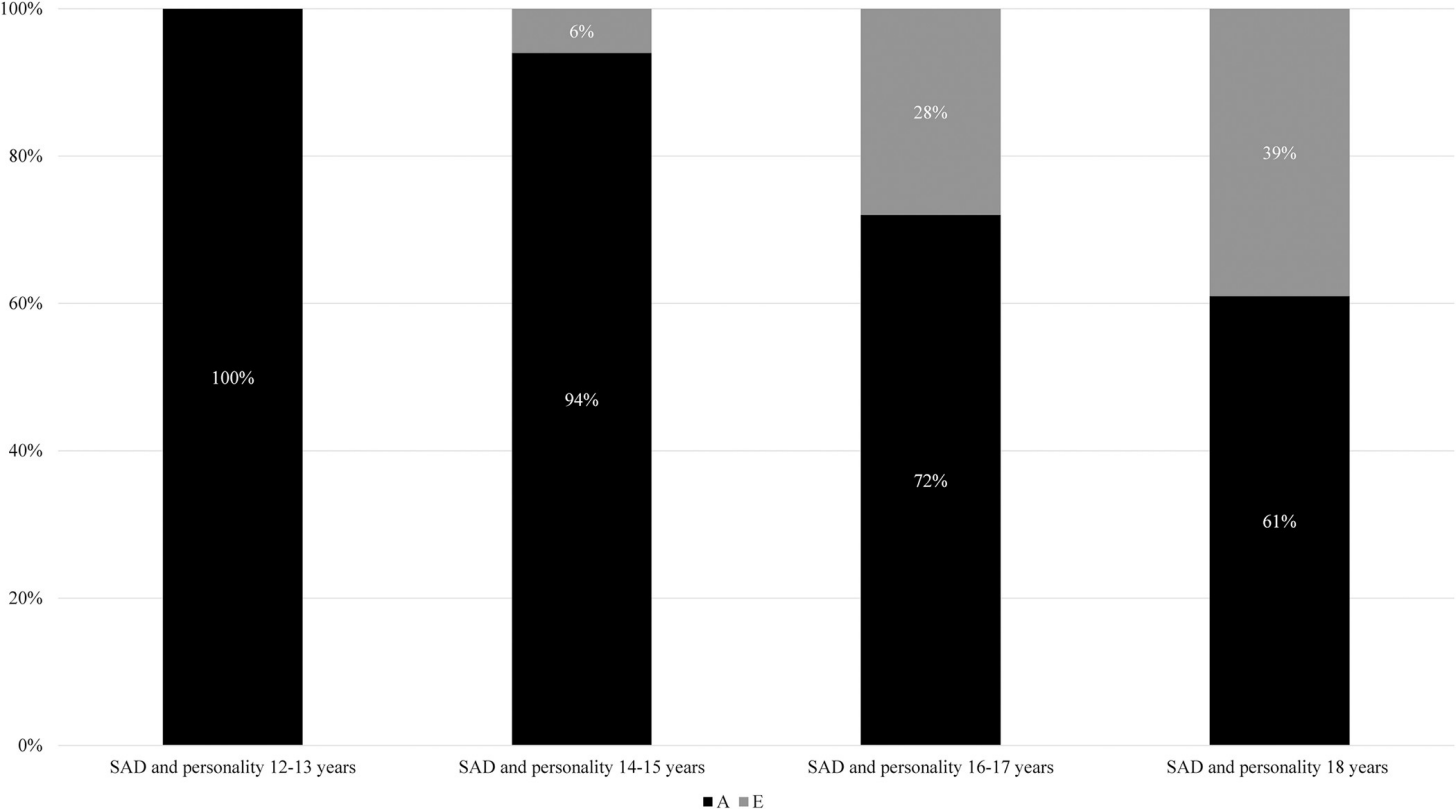
Genetic and environmental influences on the association between personality and SAD. *Note*. SAD = social anxiety disorder; A = additive genetic influences; E = non-shared environmental influences. The percentages represent the proportions of the phenotypic correlations between composite scores of personality and SAD due to genetic and environmental influences.

Given that none of the DZ correlations were greater than half the size of the MZ correlations, we fitted ADE models to data and compared them to reduced AE models. According to the AIC values, an AE model was the best fitting model for all Cholesky decompositions except the model including the composite score of personality measured at age 16–17 years. The proportions of the phenotypic correlations between personality and SAD due to genetic and environmental influences are presented in Fig 1 (standardized parameter estimates are given in S6 Table). For ease of presentation, results from the AE model are presented for all age groups in Fig 1. However, results from the full ADE models yielded similar results regarding the proportions due to genetic and environmental influences (i.e., the proportions due to non-shared environmental influences were estimated to 0%, 6%, 23% and 39% at age 12–13, 14–15, 16–17 and 18 years, respectively). Thus, the proportions due to additive genetic influences in Fig 1 could be interpreted as a broad/total percentage due to genetic influences (i.e., both additive and non-additive). In line with the pattern of cross-trait correlations, the phenotypic associations between personality and SAD were almost exclusively due to shared genetic influences when personality was measured at 12–13 years and 14–15 years. From 16–17 years, non-shared environmental influences also contributed to the association between personality and SAD.

Genetic and environmental correlations between personality and SAD are presented in Table 4. These correlations provided further support for the results presented in Fig 1. That is, all genetic correlations were high, whereas the environmental correlations were negligible when personality was measured at 12–13 years and 14–15 years. From 16–17 years of age, however, the non-shared environmental correlations between personality and SAD were statistically significant and moderate in magnitude. Genetic and environmental correlations between SAD and all individual traits are given in S7 Table.

**Table 4:** Genetic and environmental correlations.

	Genetic correlation with SAD [95% CI]	Non-Shared environmental correlation with SAD [95% CI]
Variable ^a^		
Personality 12–13 years	.75 [.54, .96]	-.05 [-.31, .22]
Personality 14–15 years	.85 [.65, 1.00]	.06 [-.14, .25]
Personality 16–17 years	.64 [.48, .80]	.32 [.12, .50]
Personality 18 years	.68 [.65, .88]	.44 [.22, .48]

*Note*. SAD = social anxiety disorder

^a^ Composite score of personality, where the personality variables were weighted relative to their importance for SAD.

**Fig 2:**
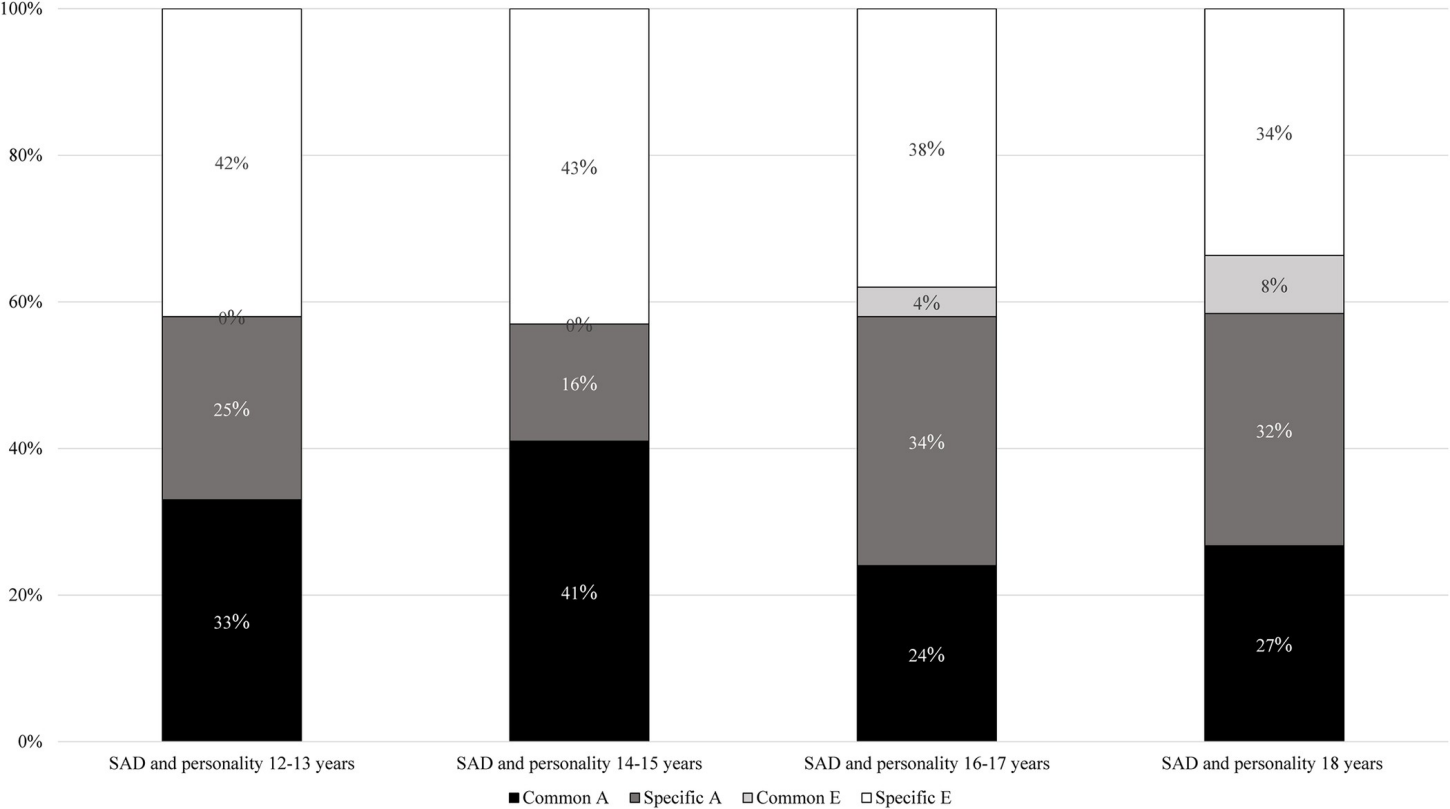
Proportion of variance in SAD that are unique to SAD and shared with personality. *Note*. SAD = social anxiety disorder; Common A = genetic factors common to both personality and SAD, Specific A = genetic factors unique to SAD; Common E = non-shared environmental influences common to both personality and SAD; Specific E = non-shared environmental influences unique to SAD.

In Fig 2, we present the genetic and environmental variance unique to SAD and the portions shared with personality at different ages throughout adolescence. The percentage of the total variance in SAD that were shared with personality due to common genetic influences was 33%–41% when personality was measured at the age of 12–15 years and 24%–27% when personality was measured at 16 years and older. If we look at only the genetic variance in SAD (i.e., ignoring the environmental variance), 57%–72% was shared with personality measured at 12–15 years (i.e., [33/33+25] and [41/41+16]). When personality was measured from 16 years and older, the percentage of the genetic variance in SAD that where shared with personality was lower, i.e., 41%–46%. Similarly, if we look at only the environmental variance in SAD (i.e., ignoring the genetic variance), SAD did not share any non-shared environmental influences with personality measured at 12–15 years. However, from 16 years of age, a small amount of the environmental variance in SAD was shared with personality (i.e., 10% and 19% when personality was measured at 16–17 years and 18 years, respectively).

## Discussion

This study examined the longitudinal associations between personality throughout adolescence and SAD in young adulthood. Despite the fact that SAD usually has its onset in early adolescence [3], the majority of studies on the relationship between personality and SAD have used adult samples. Furthermore, the overwhelming body of research has focused on the Big Five traits, and most studies are cross-sectional. The present study sought to add to the literature by examine the temporal relationship between a range of personality related variables measured at different ages throughout adolescence, and SAD in young adulthood. In line with findings from previous studies of both adult [7, 71] and adolescent [13, 16] samples, results from the present study showed that higher levels of neuroticism, lower levels of conscientiousness, and lower levels of extraversion were associated with SAD.

Of note, ‘personality’ has traditionally been called ‘temperament’ when studying children and adolescents [16]. Studies have consistently reported associations between the temperamental trait ‘behavioral inhibition’ (the tendency to be shy, quiet, and restrained in unfamiliar situations) and anxiety disorders in childhood and adolescence, especially SAD [72, 73]. In addition, ‘negative emotionality’ (the tendency to feel sadness, fear and frustration after high arousal) and ‘effortful control’ (the ability to sustain attention and inhibit behavior when appropriate) have also been linked to anxiety symptoms in childhood and adolescence [16, 74]. With respect to personality, behavioral inhibition is found to be associated with low extraversion and high neuroticism, negative emotionality shows high overlap with neuroticism, and effortful control is closely linked to conscientiousness [16]. Thus, a similar pattern of traits is found to be important for anxiety within the temperament tradition as in the personality literature, and this pattern of associations was also observed in the present study. Beyond the Big Five traits, the results showed that SAD was also associated with higher levels of loneliness, lower levels of resilience, lower self-efficacy, and lower sense of coherence throughout adolescence. Associations between SAD and these variables have been reported in previous cross-sectional studies of adolescent samples [21, 23, 26, 35].

The present study adds to the literature by showing that personality measured already in early adolescence were associated with SAD in young adulthood. More specifically, the phenotypic correlation between the composite score of personality and SAD was moderate already when personality was measured at age 12–13 years. The correlations ranged from 0.42 when personality was measured at 12–13 years of age, to 0.52 when personality and SAD was measured relatively concurrent in time. Given our finding that measures of personality may represent vulnerabilities to later development of SAD, this highlights the potential value of early assessment of personality related characteristics. This is in line with an influential meta-analysis of the relationship between the Big Five traits and common mental disorders which emphasized that more focus on personality dimensions is needed in clinical psychology [7]. The authors concluded that personality “may be helpful in directing prevention efforts, developing case conceptualizations, and making clinical prognoses” [7, p. 810]. Indeed, personality assessment may be useful for identifying adolescents at risk of developing psychopathology in general since e.g., high neuroticism and low extraversion seem to represent general psychological vulnerabilities [6, 75, 76]. In addition, personality itself should perhaps be the focus of intervention before the onset of psychopathology. For example, results from a meta-analysis suggest that personality traits seem amenable to psychological intervention, although it should be acknowledged that one does not know whether the observed changes in personality reflect ‘real’ change or reflect change in which people return to their prior baseline [77]. Nevertheless, assessment of personality may be useful to determine the focus of treatment. For example, a personality assessment may inform the clinician of a person’s strengths and vulnerabilities which may be used to guide treatment.

By using a longitudinal twin design, we were also able to examine the nature of the phenotypic associations between personality and SAD. Overall, the results showed that personality were associated with SAD mainly for genetic reasons. More specifically, the covariance between personality and SAD was exclusively due to shared genetic influences when personality was measured at 12–13 years and 14–15 years. When personality was measured later in adolescence (and closer in time to the assessment of SAD), also non-shared environmental influences contributed weakly to the covariance. That is, shared genetic influences still accounted for 2/3 of the covariance. We are not aware of any prior study that have examined the genetic and environmental contributions to the associations between personality and SAD beyond the Big Five traits. However, in line with our results, prior studies have found that shared genetic factors account for a substantial amount of the association between SAD and both neuroticism and extraversion [37–41]. Of note, genetic links have been found between personality and psychopathology in general. For example, results from a recent genetically informative study suggest that the phenotypic associations between the Big Five traits and internalizing disorders (including SAD) mainly stem from shared genetic influences [78]. More specifically, higher neuroticism and several facets from the domain of extraversion and conscientiousness seem to represent genetic susceptibility to internalizing disorders, rather than these traits having direct environmental effects on internalizing disorders.

The results in the present study contradict commonsense reasoning that would suggest that the reason why a person is the way he/she is, is because of what happened to the person in childhood or adolescence. More specifically, the results showed that environmental factors influencing both personality and SAD was negligible when personality was measured 4–5 years or more prior to the assessment of SAD. Consequently, one should be careful jumping to conclusions based on results from cross-sectional studies. Of course, this shared environmental variance will only capture environmental factors creating variance in both personality and SAD. However, it is reasonable to think that significant events that could potentially influence SAD, also contribute to variance in personality. For example, if problems at school is contributing to SAD, it is likely that this also will affect measures of personality.

It is important to have in mind that the data for the present study was based on a naturalistic study and not an intervention design. Thus, even if personality is related to SAD mainly for genetic reasons, this does not necessarily mean that interventions seeking to for example increase a person’s self-efficacy or decrease feelings of loneliness would not work. It is also important to emphasize that genetically influenced conditions may also potentially be modified, perhaps particularly through individualized/personalized therapy.

In the present study, the heritability of SAD was estimated to 59%, which is in the higher range compared to heritability estimates that have typically been reported for anxiety disorders and anxiety problems [43, 79–83]. Of note, a substantial amount of the heritability in SAD was not shared with personality, indicating that SAD is something more than personality. Surprisingly, the proportion of the genetic variance in SAD that was shared with personality was somewhat higher when personality was measured in early adolescence compared to when personality was measured at a later age and also shorter in time until the assessment of SAD. A reason may be that personality in later adolescence is influenced by “new” genes that is more unrelated to SAD.

### Limitations and strengths

The results in the present study should be considered in light of some possible limitations. First, to maximize the variance in our outcome measure, we included both clinical and subclinical scores in the measure of SAD. The results may thus not be generalized to a clinical population. However, supporting our measure of SAD, prior research has shown that individual differences between people, including differences in psychopathology, are best described as quantitative differences in degree rather than qualitative differences in kind [65]. Thus, there is no reason to expect differences in results with this procedure. A fully dimensional measure of SAD would perhaps be more appropriate as compared to our ordinal measure. However, the DSM diagnostic classification system is not developed for a continuous approach to diagnoses. Thus, the diagnostic interview did not allow for a fully dimensional measure of SAD. Second it is important to note that this is not an intervention study. Observed associations should be further studied in intervention designs for assessment of the clinical implications of the results.

Third, there are several assumptions in the classical twin design that may threaten the validity of results if violated. The equal environment assumption (EEA) states that MZ and DZ twins are exposed to the same degree of similarity of environmental factors influencing the phenotypes studied. If MZ twins experience more similar environments than DZ twins and this causes similarity in the phenotypes under study, the EEA is violated. The higher correlation in MZ twins compared to DZ twins may then be due to environmental rather than genetic influences, thus overestimating the genetic influences. However, empirical studies generally support the validity of the EEA [84–86]. Another assumption in the classical twin design is that DZ twins share half of their segregating genes. This is based on the assumption that parents do not share genes beyond what is expected by chance (i.e., random mating). If mating is not random for the phenotypes under study, DZ twins will share more than 50% genes for these phenotypes, resulting in an overestimation of the shared environmental influences and an underestimation of genetic influences. Supporting the assumption of random mating, mating is found to be low or random for personality domains [68, 87, 88].

This study also has several strengths. Using a longitudinal design allows for sequencing of predictors and outcome. The present study adds to the understanding of the role of personality in SAD by examining the longitudinal associations between a range of personality variables measured throughout adolescence and SAD in young adulthood. Furthermore, using data from twins allowed us to quantify to what extent the associations between personality and SAD were due to genetic and environmental influences. Additionally, the study is based on data from seven national birth cohorts, which strengthens the possibility of generalization of results. Participation bias may represent a threat to generalization of findings. However, participation bias is shown to be more problematic for the validity of prevalence estimates compared to estimates of associations between variables, which is the primary focus in the present study [89, 90]. In addition, the analysis of recruitment and dropout in the present study showed that attrition did not influence the heritability estimates [51].

## Conclusion

The present study demonstrated that personality measured already at the age of 12 is associated with SAD in early adulthood. More specifically, we found that higher neuroticism, lower extraversion, lower conscientiousness, higher levels of loneliness, lower levels of resilience, lower self-efficacy, and lower sense of coherence are relevant traits for the development of SAD. These traits may thus be important to pay attention to in order to identify adolescents with risk of developing SAD later in life. Furthermore, results from the present study indicate that personality is associated with SAD mainly due to shared genetic influences, rather than personality having a direct effect on SAD. Associations that are accounted for by environmental influences of a size that are practically meaningful are assumed to be more effective for prevention efforts compared to highly genetically driven associations. However, although the associations were primarily genetically mediated, this does not mean that interventions aimed to e.g., strengthen a person’s self-efficacy or reduce loneliness do not help.

## Supporting information

S1 Table The personality scales. (DOCX)

S2 Table Reliability (Cronbach’s alpha) of the personality scales. (DOCX)

S3 Table Descriptive statistics for study variables. (DOCX)

S4 Table Inter-scale correlations. (DOCX)

S5 Table Twin correlations, univariate parameter estimates, and model fit for social anxiety disorder and personality. (DOCX)

S6 Table Standardized parameter estimates from the bivariate Cholesky decomposition models. (DOCX)

S7 Table Genetic and environmental correlations between individual traits and SAD. (DOCX)
