# Supplementary material for: The relationship between personality throughout adolescence and social anxiety disorder in young adulthood. A longitudinal twin study
Source: PLoS One. 2024 Mar 13;19(3):e0299766. doi: 10.1371/journal.pone.0299766 (PMC10936778; doi:10.1371/journal.pone.0299766)
Supplement: S1 Table — (DOCX) [file pone.0299766.s001.docx]

**S1 Table****. The Personality Scales.**

**Big Five Personality**

| 1 | Talks to people easily (E) |
| --- | --- |
| 2 | Is constantly on the move (E) |
| 3 | Has difficulty making contacts (E) |
| 4 | Makes an all out effort (C) |
| 5 | Is afraid of failure (N) |
| 6 | Can use everyday things in a new way (O) |
| 7 | Quick to argue (A) |
| 8 | Has a sense of order (C) |
| 9 | Feels inferior to others (N) |
| 10 | Acts the boss (A) |
| 11 | Is quick to worry about things (N) |
| 12 | Has confidence in own abilities (N) |
| 13 | Has a broad range of interests (O) |
| 14 | Sees the sunny side of things (E) |
| 15 | Accepts authority (A) |
| 16 | Makes heavy demands on him/herself (C) |
| 17 | Says little on own initiative (E) |
| 18 | Doubs him/herself (N) |
| 19 | Finishes tasks to the very end (C) |
| 20 | Is chiefly concerned with him/herself (A) |
| 21 | Examined the way things are made (O) |
| 22 | Enjoys life (E) |
| 23 | Can concentrate on one thing for a long time (C) |
| 24 | Is quick to understand things (O) |
| 25 | Postpones troublesome things (C) |
| 26 | Works hard to get full marks (C) |
| 27 | Quick to doubt his/her own capacities (N) |
| 28 | Is interested in everything (O) |
| 29 | Grasps the meaning of things quickly (O) |
| 30 | Is easily depressed (N) |
| 31 | Has a rich imagination (O) |
| 32 | Talks about own feelings (E) |
| 33 | Feels at ease with him/herself (N) |
| 34 | Looks for adventure (E) |
| 35 | Shows a lot of jealousy (A) |
| 36 | Contributes new ideas (O) |
| 37 | Is quick to get angry (A) |
| 38 | Delivers careful and neat work (C) |
| 39 | Can go against the grain (A) |
| 40 | Takes others into account (A) |

*Note.* N = neuroticism; E = extraversion; A = agreeableness; C = conscientiousness; O = openness.

**Self-Efficacy**

| 1 | Finish homework assignments by deadlines? |
| --- | --- |
| 2 | Concentrate on school subjects? |
| 3 | Organize your schoolwork? |
| 4 | Motivate yourself to do schoolwork? |
| 5 | Resist peer pressure to do things in school that can get you into trouble? |
| 6 | Resist peer pressure to drink beer, wine or liquor? |
| 7 | Stand firm to someone who is asking to do something unreasonable or inconvenient? |
| 8 | Make and keep female friends? |
| 9 | Make and keep male friends? |
| 10 | Work in a group? |
| 11 | Express your opinions when other classmates disagree with you? |
| 12 | Deal with situations where others are annoying you or hurting you? |

**Resilience Scale**

| 1 | When I make plans, I follow through with them |
| --- | --- |
| 2 | Keeping interested in things is important to me |
| 3 | I am determined |
| 4 | My belief in myself gets me through hard times |
| 5 | I have enough energy to do what I have to do |

**Ego-Resilience Scale**

| 1 | I enjoy dealing with new and unusual situations |
| --- | --- |
| 2 | I usually succeed in making a favorable impression on people |
| 3 | I like to do new and different things |
| 4 | My daily life is full of things that keep me interested |
| 5 | I would be willing to describe myself as a pretty “strong” personality |

**Loneliness**

| 1 | I feel in tune with the people around me |
| --- | --- |
| 2 | I can find companionship when I want it |
| 3 | No one really knows me well |
| 4 | People are around me but not with me |
| 5 | I feel lonely |

**Sense of Coherence**

| 1 | Do you have the feeling that you are being treated unfairly? |
| --- | --- |
| 2 | Do you have the feeling that you are in an unfamiliar situation and don’t know what to do? |
| 3 | Do you have very mixed-up feeling and ideas? |
| 4 | Does it happen that you have feelings inside you would rather not feel? |
| 5 | How often do you have the feeling that there’s little meaning in the things you do in your daily life? |

**Delinquency**

| 1 | Had a fist fight with another person? |
| --- | --- |
| 2 | Carried a weapon on your person (a chain, knife, gun etc.)? |
| 3 | Damaged or willfully destroyed any musical instruments, sports equipment, or other school equipment? |
| 4 | Willfully damaged or destroyed public or private property that doesn’t belong to you? |
| 5 | Broken open a door or window and entered somewhere to steal something? |
| 6 | Made anonymous phone calls (without giving your name)? |
| 7 | Hung around or “fooled around” at night when you were supposed to be home? |
| 8 | Stayed away from home for more than 24 hours? |
| 9 | Missed school without a legitimate excuse? |

**Conduct Problems**

| 1 | Often has temper tantrums or hot tempers |
| --- | --- |
| 2 | Generally obedient, usually does what adults request |
| 3 | Often fights with other children or bullies them |
| 4 | Often lies or cheats |
| 5 | Steals from home, school or elsewhere |

**Impulsivity**

| 1 | I often act on the spur of the moment |
| --- | --- |
| 2 | I am more likely to be fast and careless than to be slow and plodding |
| 3 | I usually make up my mind through careful reasoning |
| 4 | People say I’m methodological and systematic in everything I do |
| 5 | I plan and organize things in detail |
| 6 | I am a cautious person |
| 7 | I often act without thinking. |
