# Supplementary material for: The relationship between personality throughout adolescence and social anxiety disorder in young adulthood. A longitudinal twin study
Source: PLoS One. 2024 Mar 13;19(3):e0299766. doi: 10.1371/journal.pone.0299766 (PMC10936778; doi:10.1371/journal.pone.0299766)
Supplement: S2 Table — (DOCX) [file pone.0299766.s002.docx]

**S2 Table. Reliability (Cronbach’s Alpha) of the Personality Scales.**

|  |  | α | | |
| --- | --- | --- | --- | --- |
| Variable | Number of items | Wave–1 | Wave–2 | Wave-3 |
| Neuroticism | 8 | .80 | .84 | .86 |
| Extraversion | 8 | .72 | .76 | .78 |
| Openness | 8 | .76 | .78 | .79 |
| Agreeableness | 8 | .71 | .72 | .72 |
| Conscientiousness | 8 | .74 | .78 | .77 |
| Self-efficacy | 12 | .79 | .80 | .79 |
| Resilience Scale | 5 | .78 | .79 | .78 |
| Ego Resilience | 5 | .76 | .78 | .78 |
| Loneliness | 5 | .78 | .81 | .84 |
| Sense of coherence | 5 | .82 | .82 | .83 |
| Delinquency | 9 | .65 | .63 | .59 |
| Conduct problems | 5 | .52 | .45 | .37 |
| Impulsivity | 7 | .61 | .65 | .73 |
