# Supplementary material for: The relationship between personality throughout adolescence and social anxiety disorder in young adulthood. A longitudinal twin study
Source: PLoS One. 2024 Mar 13;19(3):e0299766. doi: 10.1371/journal.pone.0299766 (PMC10936778; doi:10.1371/journal.pone.0299766)
Supplement: S3 Table — (DOCX) [file pone.0299766.s003.docx]

**S3 Table.** **Descriptive Statistics for Study Variables.**

|  | 12–13 years |  | 14–15 years |  | 16–17 years |  | 18 years |
| --- | --- | --- | --- | --- | --- | --- | --- |
| Variable | *M (SD)* |  | *M (SD)* |  | *M (SD)* |  | *M (SD)* |
| N | 1.37 (0.65) |  | 1.51 (0.69) |  | 1.59 (0.73) |  | 1.59 (0.76) |
| E | 2.48 (0.58) |  | 2.44 (0.59) |  | 2.42 (0.63) |  | 2.46 (0.65) |
| O | 2.22 (0.64) |  | 2.18 (0.59) |  | 2.15 (0.61) |  | 2.21 (0.63) |
| A | 2.87 (0.57) |  | 2.82 (0.56) |  | 2.80 (0.57) |  | 2.88 (0.58) |
| C | 2.43 (0.64) |  | 2.42 (0.61) |  | 2.39 (0.63) |  | 2.48 (0.60) |
| SEF | 2.93 (0.53) |  | 2.84 (0.53) |  | 2.75 (0.55) |  | 2.72 (0.53) |
| RS | 2.57 (0.69) |  | 2.61 (0.64) |  | 2.59 (0.66) |  | 2.64 (0.65) |
| ER | 2.56 (0.70) |  | 2.57 (0.65) |  | 2.57 (0.66) |  | 2.64 (0.67) |
| LON | 1.00 (0.68) |  | 1.05 (0.71) |  | 1.08 (0.75) |  | 1.07 (0.76) |
| SOC | 5.42 (1.21) |  | 5.09 (1.23) |  | 4.88 (1.30) |  | 4.95 (1.28) |
| DEL | 0.06 (0.12) |  | 0.13 (0.19) |  | 0.17 (0.22) |  | 0.15 (0.19) |
| CON | 0.28 (0.27) |  | 0.27 (0.26) |  | 0.26 (0.26) |  | 0.22 (0.22) |
| IMP | 1.00 (0.32) |  | 1.02 (0.35) |  | 1.02 (0.37) |  | 0.99 (0.39) |

*Note.* N = neuroticism; E = extraversion; O = openness; A = agreeableness; C = conscientiousness; SEF = self-efficacy; RS = Resilience Scale; ER = Ego Resilience; LON = loneliness; SOC = sense of coherence; DEL = delinquency; CON = conduct problems; IMP = impulsivity.
