# Supplementary material for: The relationship between personality throughout adolescence and social anxiety disorder in young adulthood. A longitudinal twin study
Source: PLoS One. 2024 Mar 13;19(3):e0299766. doi: 10.1371/journal.pone.0299766 (PMC10936778; doi:10.1371/journal.pone.0299766)
Supplement: S4 Table — (DOCX) [file pone.0299766.s004.docx]

**S4 Table.** **Inter-Scale Correlations.**

|  |  | Sex ^a^ | N | E | O | A | C | SEF | RS | ER | LON | SOC | DEL | CON |
| --- | --- | --- | --- | --- | --- | --- | --- | --- | --- | --- | --- | --- | --- | --- |
| 12–13 years | |  |  |  |  |  |  |  |  |  |  |  |  |  |
|  | N | .23^***^ | – |  |  |  |  |  |  |  |  |  |  |  |
|  | E | -.07^*^ | -.47^***^ | – |  |  |  |  |  |  |  |  |  |  |
|  | O | -.09^**^ | -.27^***^ | .59^***^ | – |  |  |  |  |  |  |  |  |  |
|  | A | -.02 | -.45^***^ | .16^***^ | .13^***^ | – |  |  |  |  |  |  |  |  |
|  | C | .09^*^ | -.27^***^ | .40^***^ | .53^***^ | .30^***^ | – |  |  |  |  |  |  |  |
|  | SEF | .06 | -.38^***^ | .40^***^ | .38^***^ | .34^***^ | .50^***^ | – |  |  |  |  |  |  |
|  | RS | -.09^**^ | -.41^***^ | .61^***^ | .66^***^ | .23^***^ | .60^***^ | .47^***^ | – |  |  |  |  |  |
|  | ER | -.02 | -.39^***^ | .66^***^ | .68^***^ | .22^***^ | .53^***^ | .43^***^ | .74^***^ | – |  |  |  |  |
|  | LON | .06 | .52^***^ | -.55^***^ | -.37^***^ | -.32^***^ | -.37^***^ | -.44^***^ | -.48^***^ | -.52^***^ | – |  |  |  |
|  | SOC | -.09^**^ | -.47^***^ | .15^***^ | .06 | .36^***^ | .15^***^ | .31^***^ | .18^***^ | .17^***^ | -.37^***^ | – |  |  |
|  | DEL | -.13^***^ | .15^***^ | .01 | -.04 | -.32^***^ | -.18^***^ | -.17^***^ | -.07^*^ | -.06 | .11^**^ | -.22^***^ | – |  |
|  | CON | -.04 | .31^***^ | -.12^***^ | -.14^***^ | -.53^***^ | -.30^***^ | -.32^***^ | -.21^***^ | -.16^***^ | .25^***^ | -.35^***^ | .38^***^ | – |
|  | IMP | -.05 | .01 | .12^***^ | -.07^*^ | -.21^***^ | -.40^***^ | -.17^***^ | -.10^**^ | -.02 | .01 | -.08^*^ | .19^***^ | .26^***^ |
| 14–15 years | |  |  |  |  |  |  |  |  |  |  |  |  |  |
|  | N | .30^***^ | – |  |  |  |  |  |  |  |  |  |  |  |
|  | E | -.02 | -.42^***^ | – |  |  |  |  |  |  |  |  |  |  |
|  | O | -.14^***^ | -.24^***^ | .49^***^ | – |  |  |  |  |  |  |  |  |  |
|  | A | -.02 | -.36^***^ | .03 | .03 | – |  |  |  |  |  |  |  |  |
|  | C | .10^***^ | -.20^***^ | .30^***^ | .47^***^ | .29^***^ | – |  |  |  |  |  |  |  |
|  | SEF | -.01 | -.39^***^ | .41^***^ | .37^***^ | .25^***^ | .54^***^ | – |  |  |  |  |  |  |
|  | RS | -.03 | -.39^***^ | .55^***^ | .55^***^ | .16^***^ | .58^***^ | .49^***^ | – |  |  |  |  |  |
|  | ER | -.06^*^ | -.39^***^ | .64^***^ | .58^***^ | .12^***^ | .45^***^ | .48^***^ | .71^***^ | – |  |  |  |  |
|  | LON | .04 | .52^***^ | -.54^***^ | -.25^***^ | -.24^***^ | -.29^***^ | -.48^***^ | -.44^***^ | -.47^***^ | – |  |  |  |
|  | SOC | -.20^***^ | -.55^***^ | .18^***^ | .07^**^ | .37^***^ | .19^***^ | .36^***^ | .22^***^ | .21^***^ | -.42^***^ | – |  |  |
|  | DEL | -.09^***^ | .15^***^ | .06^*^ | -.01 | -.34^***^ | -.26^***^ | -.23^***^ | -.08^**^ | -.01 | .12^***^ | -.32^***^ | – |  |
|  | CON | -.03 | .22^***^ | -.02 | -.12^***^ | -.56^***^ | -.36^***^ | -.29^***^ | -.18^***^ | -.11^***^ | .20^***^ | -.32^***^ | .44^***^ | – |
|  | IMP | -.13^***^ | -.05^*^ | .16^***^ | -.04 | -.29^***^ | -.45^***^ | -.13^***^ | -.14^***^ | .02 | -.02 | -.10^***^ | .29^***^ | .29^***^ |
| 16–17 years | |  |  |  |  |  |  |  |  |  |  |  |  |  |
|  | N | .35^***^ | – |  |  |  |  |  |  |  |  |  |  |  |
|  | E | -.03 | -.46^***^ | – |  |  |  |  |  |  |  |  |  |  |
|  | O | -.20^***^ | -.28^***^ | .46^***^ | – |  |  |  |  |  |  |  |  |  |
|  | A | -.04 | -.32^***^ | .07^**^ | .05^*^ | – |  |  |  |  |  |  |  |  |
|  | C | .07^**^ | -.17^***^ | .29^***^ | .44^***^ | .25^***^ | – |  |  |  |  |  |  |  |
|  | SEF | -.04 | -.41^***^ | .45^***^ | .36^***^ | .23^***^ | .48^***^ | – |  |  |  |  |  |  |
|  | RS | -.11^***^ | -.44^***^ | .53^***^ | .51^***^ | .20^***^ | .60^***^ | .47^***^ | – |  |  |  |  |  |
|  | ER | -.11^***^ | -.45^***^ | .67^***^ | .57^***^ | .15^***^ | .40^***^ | .45^***^ | .69^***^ | – |  |  |  |  |
|  | LON | .06^**^ | .53^***^ | -.62^***^ | -.21^***^ | -.25^***^ | -.23^***^ | -.49^***^ | -.44^***^ | -.48^***^ | – |  |  |  |
|  | SOC | -.22^***^ | -.58^***^ | .24^***^ | .10^***^ | .38^***^ | .20^***^ | .35^***^ | .31^***^ | .27^***^ | -.44^***^ | – |  |  |
|  | DEL | -.07^**^ | .10^***^ | .03 | -.05^*^ | -.28^***^ | -.28^***^ | -.21^***^ | -.16^***^ | -.02 | .13^***^ | -.23^***^ | – |  |
|  | CON | -.01 | .20^***^ | -.10^***^ | -.16^***^ | -.58^***^ | -.34^***^ | -.25^***^ | -.25^***^ | -.15^***^ | .20^***^ | -.29^***^ | .35^***^ | – |
|  | IMP | -.11^***^ | -.13^***^ | .21^***^ | -.03 | -.22^***^ | -.48^***^ | -.10^***^ | -.13^***^ | .08^***^ | -.08^***^ | -.06^*^ | .28^***^ | .27^***^ |
| 18 years | |  |  |  |  |  |  |  |  |  |  |  |  |  |
|  | N | .33^***^ | – |  |  |  |  |  |  |  |  |  |  |  |
|  | E | -.03 | -.52^***^ | – |  |  |  |  |  |  |  |  |  |  |
|  | O | -.20^***^ | -.28^***^ | .46^***^ | – |  |  |  |  |  |  |  |  |  |
|  | A | .00 | -.26^***^ | .03 | -.02 | – |  |  |  |  |  |  |  |  |
|  | C | .05^*^ | -.16 | .27^***^ | .37^***^ | .25^***^ | – |  |  |  |  |  |  |  |
|  | SEF | -.05^*^ | -.41^***^ | .45^***^ | .31^***^ | .21^***^ | .45^***^ | – |  |  |  |  |  |  |
|  | RS | -.10^***^ | -.44^***^ | .51^***^ | .50^***^ | .16^***^ | .58^***^ | .44^***^ | – |  |  |  |  |  |
|  | ER | -.08^**^ | -.49^***^ | .69^***^ | .57^***^ | .13^***^ | .37^***^ | .46^***^ | .67^***^ | – |  |  |  |  |
|  | LON | .03 | .54^***^ | -.58^***^ | -.21^***^ | -.26^***^ | -.21^***^ | -.47^***^ | -.38^***^ | -.47^***^ | – |  |  |  |
|  | SOC | -.17^***^ | -.59^***^ | .30^***^ | .09^***^ | .37^***^ | .20^***^ | .38^***^ | .32^***^ | .32^***^ | -.48^***^ | – |  |  |
|  | DEL | -.12^***^ | .07^*^ | .02 | .05 | -.32^***^ | -.27^***^ | -.18^***^ | -.11^***^ | -.02 | .06^*^ | -.25^***^ | – |  |
|  | CON | .02 | .21^***^ | -.07^*^ | -.07^*^ | -.62^***^ | -.32^***^ | -.20^***^ | -.20^***^ | -.14^***^ | .20^***^ | -.33^***^ | .36^***^ | – |
|  | IMP | -.10^***^ | -.20^***^ | .26^***^ | .05 | -.21^***^ | -.45^***^ | -.06^*^ | -.11^***^ | .12^***^ | -.12^***^ | .01 | .24^***^ | .24^***^ |

*Note.* N = neuroticism; E = extraversion; A = agreeableness; C = conscientiousness; O = openness; SEF = self-efficacy; RS = Resilience Scale; ER = Ego Resilience; LON = loneliness; SOC = sense of coherence; DEL = delinquency; CON = conduct problems; IMP = impulsivity.

^a^ Sex coded 0 = male, 1 = female. The correlation between sex and SAD was 0.05**.

^*^*p* < 0.05. ^**^*p* < 0.01. ^***^*p* < 0.001.
