# Supplementary material for: The relationship between personality throughout adolescence and social anxiety disorder in young adulthood. A longitudinal twin study
Source: PLoS One. 2024 Mar 13;19(3):e0299766. doi: 10.1371/journal.pone.0299766 (PMC10936778; doi:10.1371/journal.pone.0299766)
Supplement: S5 Table — (DOCX) [file pone.0299766.s005.docx]

**S5 Table.** **Twin Correlations, Univariate Parameter Estimates, and Model Fit for Social Anxiety Disorder and Personality.**

| Variable | rMZ [95% CI] | rDZ [95% CI] | A [95% CI] | D [95% CI] | E [95% CI] | AIC |
| --- | --- | --- | --- | --- | --- | --- |
| Social anxiety disorder | .59 [.49, .70] | .28 [.12, .40] | .47 [.00, .59] | .13 [.00, .69] | .41 [.29, .54] | 2386.47 |
|  |  |  | **.59 [.46, .70]** | **–** | **.41 [.30, .54]** | **2384.58** |
| Personality 12–13 years ^a^ | .62 [.50, .71] | .28 [.16, .40] | .43 [.00, .69] | .20 [.00, .68] | .37 [.29, .48] | -804.78 |
|  |  |  | **.61 [.51, .70]** | **–** | **.39 [30, .49]** | **-806.14** |
| Personality 14–15 years ^a^ | .48 [.38, .57] | .23 [.14, .32] | .45 [.07, .55] | .02 [.00, .42] | .53 [.45, .62] | -1592.48 |
|  |  |  | **.47 [.38, .55]** | **–** | **.53 [.45, .62]** | **-1594.48** |
| Personality 16–17 years ^a^ | .63 [.56, .70] | .10 [.01, .19] | **.00 [.00, .18]** | **.60 [.41, .66]** | **.40 [.34, .47]** | **-2024.11** |
|  |  |  | .55 [.47, .62] | – | .45 [.38, .53] | -2005.91 |
| Personality 18 years ^a^ | .48 [.37, .58] | .13 [.02, .23] | **.00 [.00, .44]** | **.46 [.00, .55]** | **.54 [.45, .64]** | **-973.33** |
|  |  |  | .42 [.32, .51] | – | .58 [.49, .68] | -971.63 |

*Note.* rMZ = correlation within monozygotic twin pairs; rDZ = correlation within dizygotic twin pairs; A = additive genes; D = non-additive genes; E = non-shared environment; AIC = Akaike’s information criterion. Polychoric correlations are reported for social anxiety disorder and Pearson correlations are reported for the composite scores. Full ADE models are presented in the first row for each variable, followed by reduced AE models. The best fitting model are indicated in bold. ^a^ = Composite score of personality, where the personality variables were weighted relative to their importance for SAD.
