# Supplementary material for: The relationship between personality throughout adolescence and social anxiety disorder in young adulthood. A longitudinal twin study
Source: PLoS One. 2024 Mar 13;19(3):e0299766. doi: 10.1371/journal.pone.0299766 (PMC10936778; doi:10.1371/journal.pone.0299766)
Supplement: S6 Table — (DOCX) [file pone.0299766.s006.docx]

**S6 Table.** **Standardized Parameter Estimates from the Bivariate Cholesky Decomposition Models.**

| Model ^a^ | A_11_ | A_12_ | A_22_ | E_11_ | E_12_ | E_22_ |
| --- | --- | --- | --- | --- | --- | --- |
| Personality 12-13 years and SAD | .78 [.72, .83] | .57 [.41, .72] | .50 [.19, .67] | .62 [.55, .70] | -.03 [-.20, .14] | .65 [.56, .74] |
| Personality 14-15 years and SAD | .69 [.63, .75] | .64 [.49, .78] | .40 [.00, .59] | .72 [.67, .78] | .04 [-.09, .17] | .65 [.56, .75] |
| Personality 16-17 years and SAD | .75 [.69, .79] | .49 [.47, .50] | .58 [.43, .59] | .66 [.61, .72] | .21 [.18, .23] | .62 [.52, .63] |
| Personality 18 years and SAD | .64 [.56, .71] | .52 [.48, .54] | .56 [.35, .59] | .76 [.70, .83] | .28 [.25, .31] | .58 [.47, .60] |

*Note.* 95% CI in brackets. SAD = social anxiety disorder; A = additive genetic influences; E = non-shared environmental influences; _11_ = Genetic and environmental influences on personality; _12_ = Genetic and environmental influences on personality, contributing to variance in SAD; _22_ = Genetic and environmental influences unique to SAD. ^a^ The first variable in each model is the composite score of personality, where the personality variables were weighted relative to their importance for SAD. The second variable in each model is SAD.
