# Supplementary material for: The relationship between personality throughout adolescence and social anxiety disorder in young adulthood. A longitudinal twin study
Source: PLoS One. 2024 Mar 13;19(3):e0299766. doi: 10.1371/journal.pone.0299766 (PMC10936778; doi:10.1371/journal.pone.0299766)
Supplement: S7 Table — (DOCX) [file pone.0299766.s007.docx]

**S7 Table. Genetic and Environmental Correlations between Individual Traits and SAD.**

|  | | Genetic correlation with SAD [95% CI] ^a^ | Non-Shared environmental correlation with SAD [95% CI] |
| --- | --- | --- | --- |
| Neuroticism | |  |  |
|  | 12–13 years | **.74 [.41, 1.00]** | -.19 [-.44, .09] |
|  | 14–15 years | **.71 [.49, .93]** | .06 [-.13, .25] |
|  | 16–17 years | **.41 [.21, .60]** | **.28 [.08, .46]** |
|  | 18 years | **.52 [.28, .77]** ^*^ | **.30 [.05, .52]** |
| Extraversion | |  |  |
|  | 12–13 years | **-.48 [-.74, -.21]** ^*^ | -.16 [-.41, .10] |
|  | 14–15 years | **-.66 [-.87, -.45]** | -.03 [-.22, .18] |
|  | 16–17 years | **-.56 [-.73, -.39]** ^*^ | **-.21 [-.41, -.01]** |
|  | 18 years | **-.61 [-.82, -.41]** ^*^ | **-.39 [-.58, -.17]** |
| Openness | |  |  |
|  | 12–13 years | **-.62 [-.96, -.30]** | .07 [-.18, .31] |
|  | 14–15 years | **-.39 [-.61, -.16]** | .03 [-.18, .23] |
|  | 16–17 years | **-.22 [-.43, -.01]** ^*^ | -.02 [-.24, .19] |
|  | 18 years | -.20 [-.41, .02] | -.18 [-.39, .05] |
| Conscientiousness | |  |  |
|  | 12–13 years | **-.57 [-.88, -.28]** ^*^ | .08 [-.19, .34] |
|  | 14–15 years | **-.40 [-.61, -.19]** ^*^ | .07 [-.15, .27] |
|  | 16–17 years | **-.25 [-.45, -.04]** | -.08 [-.27, .12] |
|  | 18 years | -.13 [-.35, .09] ^*^ | **-.26 [-.47, -.02]** |
| Self-efficacy | |  |  |
|  | 12–13 years | **-.50 [-.74, -.25]** | .02 [-.24, .27] |
|  | 14–15 years | **-.47 [-.67, -.28]** | .14 [-.07, .34] |
|  | 16–17 years | **-.44 [-.62, -.25]** ^*^ | -.14 [-.34, .07] |
|  | 18 years | **-.27 [-.50, -.04]** | **-.27 [-.48, -04]** |
| Resilience Scale | |  |  |
|  | 12–13 years | **-.39 [-.68, -.10]** ^*^ | -.21 [-.47, .09] |
|  | 14–15 years | **-.40 [-.61, -.19]** ^*^ | -.06 [-.26, .15] |
|  | 16–17 years | **-.30 [-.52, -.07]** ^*^ | -.19 [-.39, .02] |
|  | 18 years | **-.33 [-.57, -.09]** ^*^ | -.20 [-.43, .05] |
| Ego Resilience | |  |  |
|  | 12–13 years | **-.50 [-.81, -.19]** | -.08 [-.36, .20] |
|  | 14–15 years | **-.52 [-.73, -.32]** ^*^ | -.02 [-.23, .18] |
|  | 16–17 years | **-.34 [-.56, -.12]** ^*^ | **-.30 [-.50, -.10]** |
|  | 18 years | **-.52 [-.77, -.27]** | -.22 [-.43, .00] |
| Loneliness | |  |  |
|  | 12–13 years | **.61 [.36, .87]** | .04 [-.20, .27] |
|  | 14–15 years | **.58 [.38, .78]** | .02 [-.17, .21] |
|  | 16–17 years | **.49 [.30, .68]** | .17 [-.03, .36] |
|  | 18 years | **.50 [.25, 76]** | **.23 [.01, .43]** |
| Sense of coherence | |  |  |
|  | 12–13 years | **-.66 [-.99, -.36]** | .21 [-.02, .42] |
|  | 14–15 years | **-.34 [-.55, -.12]** ^*^ | -.16 [-.35, .05] |
|  | 16–17 years | **-.42 [-.65, -.18]** | **-.22 [-.41, -.03]** |
|  | 18 years | **-.40 [-.64, -.16]** ^*^ | -.23 [-.47, .04] |
| Delinquency ^b^ | |  |  |
|  | 12–13 years | -.02 [-.05, .01] ^*^ | .24 [-.03, .48] |
|  | 14–15 years^*^ | **.22 [.19, .45]** ^*^ | -.08 [-.27, .12] |
|  | 16–17 years^*^ | .06 [-.14, .10] ^*^ | .13 [-.08, .34] |
|  | 18 years^*^ | **.17 [.14 .20]** | **-.09 [-.32, -.04]** |
| Conduct problems ^b^ | |  |  |
|  | 12–13 years^*^ | -.12 [-.48, .23] | .17 [-.11, .43] |
|  | 14–15 years | **.52 [.47, .83]** | -.12 [-.30, .07] |
|  | 16–17 years^*^ | .17 [-.06, .41] ^*^ | -.05 [-.27, .17] |
|  | 18 years^*^ | **.18 [.15, .22]** ^*^ | -.01 [-.05, .24] |
| Impulsivity ^b^ | |  |  |
|  | 12–13 years^*^ | .13 [-.24, .52] ^*^ | -.14 [-.39, .12] |
|  | 14–15 years^*^ | -.07 [-.34, .20] ^*^ | -.07 [-.28, .14] |
|  | 16–17 years | **-.45 [-.72, -.19]** ^*^ | .17 [-.05, .38] |
|  | 18 years | **-.30 [-.56, -.03]** | -.08 [-.28, .12] |

*Note.* SAD = social anxiety disorder. Statistically significant correlations are indicated in bold. Genetic and environmental correlations between SAD and agreeableness are not shown because all phenotypic correlations between these traits were non-significant.

^a^ The presence of an asterisk (^*^) after the genetic correlation indicates a non-additive genetic correlation, while a genetic correlation without an asterisk refers to an additive genetic correlation. ^b^ Some of the phenotypic correlations between SAD and delinquency, conduct problems and impulsivity were statistically non-significant. However, for completeness, we report genetic and environmental correlations for all age groups. The presence of an asterisk (^*^) after the age group indicates a non-significant phenotypic correlation between SAD and the given trait.
